# Mapping deep endometriosis in patients with ovarian endometriomas according to the #Enzian classification: a single-center retrospective analysis

**DOI:** 10.3389/fmed.2025.1626445

**Published:** 2025-07-25

**Authors:** Vimee Bindra, Antoine Naem, P. Swetha, Joelle Kahla, Archana Reddy, Antonio Simone Laganà, Andrea Etrusco, Dimitrios Andrikos, Gaetano Riemma, Vittorio Agrifoglio, Alin Constantin, Rudy Leon De Wilde, Harald Krentel

**Affiliations:** ^1^Endometriosis Centre, Apollo Health City, Hyderabad, India; ^2^Faculty of Mathematics and Computer Science, University of Bremen, Bremen, Germany; ^3^Central Surgical Unit, Hospital St. Joseph Stift, Bremen, Germany; ^4^Faculty of Medicine of Damascus University, Damascus, Syria; ^5^Unit of Obstetrics and Gynecology, “Paolo Giaccone” Hospital, Department of Health Promotion, Mother and Child Care, Internal Medicine and Medical Specialties (PROMISE), University of Palermo, Palermo, Italy; ^6^Department of Obstetrics, Gynecology, Gynecologic Oncology and Senology, Bethesda Hospital Duisburg, Duisburg, Germany; ^7^Obstetrics and Gynecology Unit, Department of Woman, Child and General and Specialized Surgery, University of Campania "Luigi Vanvitelli", Naples, Italy; ^8^Department of Gynecology, Obstetrics and Reproduction Medicine, Saarland University, Homburg, Germany; ^9^University Hospital for Gynecology, Carl von Ossietzky University, Oldenburg, Germany

**Keywords:** anatomy, endometriosis, endometrioma, #Enzian, deep endometriosis, distribution, pathogenesis

## Abstract

**Objective:**

Endometriosis is a multifactorial disease that affects mainly women of reproductive age. It is unclear whether each form of pelvic endometriosis is an independent entity and whether a spatial relationship between all three endometriosis forms exists. We aimed with this research to examine the distribution of deep endometriosis in patients with ovarian endometriomas. We also aimed to assess the influence of unilateral and bilateral ovarian endometriomas on the distribution of deep endometriotic nodules.

**Methods:**

This is a retrospective single-center cohort study that included all patients with histologically-proven ovarian endometriomas. The #Enzian classification was used to classify endometriosis. The distribution of deep endometriosis in patients with endometriomas was assessed.

**Results:**

A total of 106 out of 121 patients with ovarian endometriomas had coexisting deep endometriosis (87.6%). Bilateral endometriomas were more common than unilateral ones. There was a statistically significant correlation between ipsilateral ovarian endometriomas and same-sided deep endometriosis of the pelvic compartment B according to the #Enzian classification. Patients with bilateral ovarian endometriomas had deep endometriosis of the left #Enzian B pelvic compartment and rectovaginal septum at a higher frequency than patients with unilateral endometriomas.

**Conclusion:**

Deep Endometriosis coexists with unilateral or bilateral ovarian endometriomas in the great majority of patients. Bilateral endometriomas are associated with higher frequencies of deep endometriosis in the left #Enzian B compartment and rectovaginal septum (#Enzian A). In order to avoid incomplete surgeries and complications, patients with endometriomas should be carefully screened for deep endometriosis of the pelvis using transvaginal ultrasound or MR imaging.

## 1 Introduction

Endometriosis is defined by the presence of endometrium-like glands and/ or stroma out of the uterus (1). Endometriosis affects mainly patients of reproductive age but it can be encountered in patients of all age groups (1, 2). The exact prevalence of endometriosis is unknown precisely but it is thought to affect around 10% of women of childbearing age (3, 4). Endometriosis is mainly an intra-pelvic disease (5), which means that the endometriotic lesions are most frequently encountered in the pelvis as either peritoneal endometriosis (also known as superficial endometriosis), ovarian endometriomas, and deep endometriotic nodules (6). Deep endometriosis was reported to affect 25.4% of endometriosis patients (7), 17.2% of subfertile patients (8), and 1–2% of the general population (9). Deep Endometriosis had been historically defined as any endometriotic lesion infiltrating the peritoneum for more than 5 mm (10). More recently, the definition of deep endometriosis has been modified to involve any endometriotic tissue in the abdominopelvic region extending on or under the peritoneum (11).

The ovaries are the most common site of endometriosis (12, 13). Nevertheless, deep endometriosis most frequently involves the uterosacral ligament (14). Less commonly, bowel endometriosis occurs in 8–12% of cases (15), while bladder and ureteric endometriosis are encountered in around 6 and 1.7% of cases, respectively (16, 17).

The distribution of endometriosis within the body probably gives an impression about its origin. Furthermore, it would be interesting to see how different types of the disease interact with each other. Furthermore, understanding the distribution of endometriosis will help better detect and treat the disease. Therefore, we aim with this research to investigate the distribution of different endometriotic lesions in the presence of ovarian endometriomas. We incorporated the #Enzian classification in our methods owing to our belief that it offers a standardized way of categorizing and reporting all types and localizations of endometriosis (18).

## 2 Materials and methods

This is a retrospective single-center cohort study that included all patients who were admitted to the endometriosis care center at Apollo Hospitals (Hyderabad, India) between the 1st of April 2021 and the 31st of October 2022 for surgical management of endometriosis.

The study protocol was reviewed and approved by an independent ethical review board of Apollo Hospitals (IRB approval No: AHJ-C-S-015/10-22, 22.10.2022). Written informed patient consent was obtained from all participants regarding the study’s procedures, anonymized data collection and analysis for research purposes. The conduct of the study adhered to the ethical standards of the Declaration of Helsinki (1964) and the guidelines of the Committee on Publication Ethics (COPE). This study is reported in accordance with the Reporting of studies Conducted using Observational Routinely-collected health Data (RECORD) statement (19), made available through the Enhancing the Quality and Transparency of Health Research (EQUATOR) network.^1^

The purpose of this study is to investigate the distribution of endometriosis in patients with ovarian endometriomas. We also investigated whether the lesions’ distribution may be influenced by the laterality of the ovarian endometriomas and by the presence of bilateral endometriomas. Ovarian endometriomas and deep endometriosis were diagnosed preoperatively by means of Transvaginal Ultrasonography (TVUS) and Magnetic Resonance Imaging (MRI), or both. Adenomyosis was diagnosed sonographically according to the Morphological Uterus Sonographic Assessment (MUSA) criteria (20). We included in this study all patients with pathologically-confirmed ovarian endometriomas and patients who did not receive a previous surgical treatment for ovarian endometriomas or deep endometriosis. We excluded from this study pregnant patients and those who had concomitant malignant pathologies or congenital uterine anomalies. Patients whose histopathology examination ruled out ovarian endometriomas, patients who underwent unilateral or bilateral salpingo-oophorectomy or hysterectomy, or those who refused to participate in the study were also excluded. All the included patients were operated by a single experienced endometriosis surgeon (V.B) via laparoscopic or robotic-assisted laparoscopic surgery. A standardized surgical approach was followed in all operated patients. All peritoneal endometriotic lesions were excised. Ovarian endometriomas were treated by either cystectomy or sclerotherapy. When sclerotherapy was performed, a biopsy was taken from the endometrioma’s margin at the site of the puncture for histological confirmation. In cases of deep endometriosis of the uterosacral ligaments, cardinal ligaments, and/or the pelvic sidewalls (#Enzian B), the ureters were freed through ureterolysis in cases of extrinsic involvement. No cases of intrinsic ureteric involvement were encountered in this cohort of patients. Rectal endometriosis was treated by rectal shaving, discoid excision, or segmental rectal resection.

All patients underwent a tubal patency test through intrauterine injection of methylene blue. The #Enzian classification of endometriosis was established intraoperatively (18). The #Enzian classification is a visual classification of endometriosis, where each letter of the equation refers to an anatomical location of the endometriotic lesions, while the numbers nearby indicate the lesions´ size. The locations that the #Enzian classification refers to are namely: the peritoneum (P), the ovaries (O), the Fallopian tubes (T), the rectovaginal septum (A), the pelvic sidewalls (B), the rectum (C), the intestines (FI), the ureters (FU), adenomyosis of the uterus (FA), and opens the possibility to documenting endometriosis in less common locations like the diaphragm (F Diaphragm). Only lesions with postoperative histopathologic confirmation were taken into account and the classification coding was verified on that basis.

Data regarding the patients’ demographics, medical and gynecologic history, symptomatology, and surgical findings including their surgical #Enzian classification were collected in a questionnaire that was designed specifically for this study.

Descriptive statistics were used for the data analysis. The normally distributed continuous variables were expressed as means ± Standard Deviation (SD). Continuous variables that were not normally distributed were expressed as medians with their Interquartile Range (IQR). The data normality was examined through the Schapiro-Wilk test. Categorical variables were expressed as frequencies and valid percentages. Means were compared through either the two-sided independent T-test or the Mann Whitney U test, depending on their distribution. Categorical variables were compared through the Chi-square (X^2^) test or Fisher’s Exact test as appropriate. We tested whether the side of unilateral ovarian endometriomas correlates with the side of deep pelvic endometriosis of the sidewalls by comparing the ratio of patients who had unilateral ovarian endometriomas and contralateral versus ipsilateral one-sided deep endometriosis of the pelvic sidewalls through the Chi-square test. The significance level was set at *p* < 0.050. The statistical analysis was performed using the Statistical Package for Social Sciences (SPSS) software, version 25.0 (SPSS, Chicago, IL, United States).

## 3 Results

### 3.1 General characteristics

A total of 121 patients presented with unilateral or bilateral ovarian endometriomas during the study period. The mean age at presentation was 31.88 ± 5.8 years old. Most of the included patients were younger than 35 years old (71.1%). The mean Body Mass Index (BMI) of the included sample was 23.94 ± 3.5 Kg/m2. The median age at menarche was 13 years old (IQR = 1). Although only 36.4% of our patients were infertile, most of the included patients were nulliparous (77.7%).

Dysmenorrhea was reported by 119 patients (98.3%). The median score of the Visual Analog Scale (VAS) for dysmenorrhea was 7 (IQR = 1). Dyschezia, Dyspareunia, and constipation were the most frequently reported symptoms as they were reported by 41.3, 38, and 35.5% of patients, respectively. Non-cyclic pelvic pain was reported by 29 patients only (24%). The general characteristics and symptomatology of our cohort is presented in Table 1.

**Table 1:** The general characteristics and symptomatology of the included participants.

Characteristics (*n* = 121)	
Age (years)	31.88 ± 5.78
BMI (Kg/m^2^)	23.94 ± 3.54
Age at menstruation (years)	13 (IQR = 1)
Menstruation duration (days)	4 (IQR = 1)
Regularity of the menstrual cycle (%)	
Regular	105 (86.8%)
Irregular	16 (13.2%)
Menstrual flow (%)	
Normal	84 (69.4%)
Menorrhagia	37 (30.6%)
Medical and gynecologic history	
Parity (%)	
Nulliparous	94 (77.7%)
Parous	27 (22.3%)
Miscarriage (%)	12 (9.9%)
Infertility (%)	44 (36.4%)
Primary infertility	30 (68.2%)
Secondary infertility	14 (31.8%)
Surgical history (%)	26 (21.5%)
Diagnostic laparoscopy (%)	7 (5.8%)
Hormonal treatment (%)	52 (43%)
Oral contraceptive pills	22 (18.2%)
Dienogest	32 (26.4%)
GnRH agonists	19 (15.7%)
Mifepristone	1 (0.8%)
Mirena	1 (0.8%)
Duration of treatment (months)	9 (IQR = 6)
CA-125 (IU/mL)	52.85 (IQR = 93.1)
AMH (ng/mL)	2.45 (IQR = 2.86)
Symptoms	
Dysmenorrhea (%)	119 (98.3%)
VAS score	7 (IQR = 1)
Non cyclic pain (%)	29 (24%)
Dyspareunia (%)	46 (38%)
Diarrhea (%)	16 (13.2%)
Dyschezia (%)	50 (41.3%)
Constipation (%)	43 (35.5%)
Hematochezia (%)	17 (14%)
Dysuria (%)	14 (11.6%)
Urinary frequency (%)	9 (7.4%)
Urinary incontinence (%)	5 (4.1%)
Flank pain (%)	27 (22.3%)
Fatigue (%)	35 (28.9%)
Vomiting (%)	19 (15.7%)
Headache (%)	22 (18.2%)
Low back pain (%)	48 (39.7%)
Leg pain (%)	47 (38.8%)

BMI, Body Mass Index; GnRH, Gonadotropins-Releasing Hormone; CA, Cancer Antigen; AMH, Anti-Müllerian Hormone; VAS, Visual Analog Scale.

### 3.2 Unilateral versus bilateral ovarian endometriomas

The great majority of patients presenting with either unilateral or bilateral ovarian endometriomas had coexisting deep endometriosis (*n* = 106, 87.6%). Table 2 represents the #Enzian classification with the relevant surgical findings of the included sample. An overall of 47 (38.8%) patients had unilateral ovarian endometriomas and 74 patients had bilateral ovarian endometriomas (61.2%). Table 3 reports the general characteristics and symptomatology of both study groups. The mean age at presentation of patients with unilateral endometriomas was 32.57 ± 6.3 years, while the mean age of patients presenting with bilateral endometriomas was 31.43 ± 5.4 years (*p* = 0.29). The age at menarche, the regularity of the menstrual cycle, the menstruation duration, and the menstrual flow were comparable between both groups (*p* > 0.05). The infertility rate along with its subtypes and preoperative AMH levels were comparable between patients with unilateral and bilateral ovarian endometriomas (*p* > 0.05). Nonetheless, hormonal treatment was more common among patients with bilateral than unilateral ovarian endometriomas (52.7% vs. 27.7%, *p* = 0.008). The presence of dysmenorrhea and its intensity did not differ significantly between both groups (*p* > 0.05). Although it did not reach the significance level, dyspareunia was more commonly reported by patients with bilateral ovarian endometriomas than patients with unilateral endometriomas (44.6% vs. 27.7%, *p* = 0.08). Dyschezia, Constipation, and other symptoms were also comparable between both groups (*p* > 0.05).

**Table 2:** The detailed #Enzian classification and related surgical findings of the included cohort.

#Enzian (*n* = 121)	
#Enzian P (%)	43 (35.5%)
P1	8 (6.6%)
P2	19 (15.7%)
P3	16 (13.2%)
#Enzian O (Left) (%)	102 (84.3%)
O1	24 (19.8%)
O2	52 (43%)
O3	26 (21.5%)
Left endometrioma size (cm)	4 (IQR = 4)
#Enzian O (Right) (%)	93 (77.5%)
O1	32 (26.7%)
O2	44 (36.7)
O3	17 (14.2%)
Right endometrioma size (cm)	4 (IQR = 3)
#Enzian T (Left) (%)	73 (60.3%)
T1	7 (9.6%)
T2	17 (23.3%)
T3	49 (67.1%)
#Enzian T (Right) (%)	58 (47.9%)
T1	8 (13.8%)
T2	16 (27.6%)
T3	34 (58.6%)
#Enzian A (%)	85 (70.2%)
A1	7 (8.2%)
A2	27 (31.8%)
A3	51 (60%)
#Enzian B (Left) (%)	88 (72.7%)
B1	7 (8%)
B2	75 (85.2%)
B3	6 (6.8%)
#Enzian B (Right) (%)	83 (68.6%)
B1	11 (13.3%)
B2	66 (79.5%)
B3	6 (7.2%)
#Enzian C (%)	42 (34.7%)
C1	2 (4.8%)
C2	21 (50%)
C3	19 (45.2%)
Size of Rectal Nodule (cm)	2 (IQR = 2)
#Enzian FA (%)	46 (38%)
#Enzian FU (Left) (%)	3 (2.5%)
#Enzian FU (Right) (%)	1 (0.8%)
#Enzian FI (%)	5 (4.1%)
Number of Resected Specimens	3.66 ± 1.72

IQR, Interquartile range.

**Table 3:** Comparison between the general characteristics and symptomatology of patients with unilateral and bilateral ovarian endometriomas.

Characteristics	Unilateral endometrioma (*n* = 47)	Bilateral endometriomas (*n* = 74)	*p* value
Age (years)	32.57 ± 6.3	31.43 ± 5.4	0.29
BMI (Kg/m^2^)	23.5 ± 3.8	24.22 ± 3.4	0.28
Age at Menarche (years)	13 (IQR = 1)	13 (IQR = 1)	0.71
Menstruation duration (days)	4 (IQR = 1)	4 (IQR = 1)	0.69
Menstrual cycle regularity (%)			
Regular	42 (89.4%)	63 (85.1%)	0.59
Irregular	5 (10.6%)	11 (14.9%)	
Menstrual flow (%)			
Normal	32 (68.1%)	52 (70.3%)	0.84
Menorrhagia	15 (31.9%)	22 (29.7%)	
Medical and gynecologic history			
Infertility (%)	14 (29.8%)	30 (40.5%)	0.25
Primary	10 (71.4%)	20 (66.7%)	1.00
Secondary	4 (28.6%)	10 (33.3%)	
Surgical History (%)	10 (21.3%)	16 (21.6%)	1.00
Hormonal treatment (%)	13 (27.7%)	39 (52.7%)	**0.008**
Duration of treatment (months)	12 (IQR = 17)	8 (IQR = 6.25)	0.48
CA-125 (IU/mL)	38 (IQR = 50)	60 (IQR = 112.11)	0.11
AMH (ng/mL)	3.30 (IQR = 1.87)	2.04 (IQR = 2.99)	0.09
Symptoms			
Dysmenorrhea (%)	46 (97.9%)	73 (98.6%)	1.00
VAS score	8 (IQR = 1)	7 (IQR = 1)	0.37
Non cyclic pain (%)	14 (29.8%)	15 (20.3%)	0.28
Dyspareunia (%)	13 (27.7%)	33 (44.6%)	0.08
Diarrhea (%)	7 (14.9%)	9 (12.2%)	0.78
Dyschezia (%)	15 (31.9%)	35 (47.3%)	0.13
Constipation (%)	15 (31.9%)	28 (37.8%)	0.56
Hematochezia (%)	3 (6.4%)	14 (18.9%)	0.06
Dysuria (%)	4 (8.5%)	10 (13.5%)	0.56
Urinary frequency (%)	2 (4.3%)	7 (9.5%)	0.48
Urinary incontinence (%)	1 (2.1%)	4 (5.4%)	0.65
Flank pain (%)	10 (21.3%)	17 (23%)	1.00
Fatigue (%)	12 (25.5%)	23 (31.1%)	0.54
Vomiting (%)	6 (12.8%)	13 (17.6%)	0.61
Headache (%)	8 (17%)	14 (18.9%)	0.86

BMI, Body Mass Index; CA, Cancer Antigen; AMH, Anti-Müllerian Hormone; VAS, Visual Analog Scale. Values written in bold indicate statistical significance.

The diagnosis of peritoneal endometriosis (#Enzian P) was comparable between patients with unilateral and bilateral ovarian endometriomas (36.2% vs. 35.1%, *p* = 1). Right-sided tubal involvement (#Enzian T) was more commonly encountered in patients with bilateral ovarian endometriomas (55.4% vs. 36.2%, *p* = 0.043), unlike the left-sided tubal involvement, which was comparable between both study groups. The involvement of the rectovaginal septum was more commonly seen in patients with bilateral ovarian endometriomas in comparison with those with unilateral endometriomas (77% vs. 59.6%, *p* = 0.045). Similarly, left-sided #Enzian B was more common in patients with bilateral endometriomas (82.4% vs. 57.4%, *p* = 0.003). Right-sided #Enzian B, rectal endometriosis (#Enzian C), and adenomyosis (#Enzian FA) were comparable between both groups. It should be also noted that the subgroup categorization according to the #Enzian classification of peritoneal and deep endometriosis did not differ significantly between the groups (*p* > 0.05). The intraoperative findings along with the surgical #Enzian classification of patients with unilateral and bilateral ovarian endometriomas are presented in Table 4.

**Table 4:** A comparison between the distribution of endometriosis according to the #Enzian classification in patients with unilateral and bilateral ovarian endometriomas.

#Enzian classification	Unilateral endometrioma (*n* = 47)	Bilateral endometriomas (*n* = 74)	*p*-value
#Enzian P (%)	17 (36.2%)	26 (35.1%)	1.00
P1	4 (23.5%)	4 (15.4%)	0.84
P2	7 (41.2%)	12 (46.2%)	
P3	6 (35.3%)	10 (38.5%)	
#Enzian T (Left) (%)	24 (51.1%)	49 (66.2%)	0.13
T1	2 (8.3%)	5 (10.2%)	0.68
T2	4 (16.7%)	13 (26.5%)	
T3	18 (75%)	31 (63.3%)	
#Enzian T (Right) (%)	17 (36.2%)	41 (55.4%)	**0.04**
T1	3 (17.6%)	5 (12.2%)	0.47
T2	6 (35.3%)	10 (24.4%)	
T3	8 (47.1%)	26 (63.4%)	
#Enzian A (%)	28 (59.6%)	57 (77%)	**0.045**
A1	3 (10.7%)	4 (7%)	0.42
A2	11 (39.3%)	16 (28.1%)	
A3	14 (50%)	37 (64.9%)	
#Enzian B (Left) (%)	27 (57.4%)	61 (82.4%)	**0.003**
B1	0 (0%)	7 (11.5%)	0.21
B2	25 (92.6%)	50 (82%)	
B3	2 (7.4%)	4 (6.6%)	
#Enzian B (Right) (%)	31 (66%)	52 (70.3%)	0.69
B1	4 (12.9%)	7 (13.5%)	0.84
B2	24 (77.4%)	42 (80.8%)	
B3	3 (9.7%)	3 (5.8%)	
#Enzian C (%)	12 (25.5%)	30 (40.5%)	0.12
C1	1 (8.3%)	1 (3.3%)	0.29
C2	4 (33.3%)	17 (56.7%)	
C3	7 (58.3%)	12 (40%)	
Rectal nodule size (cm)	3 (IQR = 1.75)	2 (IQR = 2)	0.31
#Enzian FA (%)	20 (42.6%)	26 (35.1%)	0.45
#Enzian FU (%)			1.00
Left	1 (2.1%)	2 (2.7%)	
Right	0 (0%)	1 (1.4%)	
#Enzian FI (%)	1 (2.1%)	4 (5.4%)	0.65
Number of Resected Specimens	3.23 ± 1.4	4.16 ± 1.5	**0.001**

IQR, Interquartile range. Values written in bold indicate statistical significance.

Finally, the patients’ characteristics, symptomatology, and distribution of endometriosis did not differ significantly between patients with and without adenomyosis (data not shown).

## 4 Discussion

The debate of whether the different types of endometriosis are correlated in terms of the pathogenesis and the anatomical distribution remains open. In this study, we demonstrated that ovarian endometriomas most often coexist with deep endometriosis, unlike peritoneal endometriosis which coexists in around one-third of the patients. Our results also emphasized the left-sided predominance of ovarian endometriomas and deep endometriosis of the uterosacral ligament, cardinal ligament, and pelvic side wall. Interestingly, deep endometriosis of the #Enzian B compartment and ovarian endometriomas correlated spatially, since left-sided ovarian endometriomas coexist more frequently with left-sided deep endometriosis and right-sided endometriomas coexist more frequently with right-sided deep endometriosis.

Those results are of paramount importance in the diagnostic and therapeutic aspects of endometriosis because they demonstrated that the great majority of patients with ovarian endometriomas have coexisting deep endometriosis.

To the best of our knowledge, there is only one study that examined the distribution of deep endometriosis in correlation with ovarian endometriomas. Kwok et al. (21) investigated the distribution of deep endometriosis in patients with ovarian endometriomas. In line with our results, left-sided ovarian endometriomas were found to be more common than right-sided endometriomas. Moreover, deep endometriotic lesions were more frequently encountered on the left than the right body side with the uterosacral ligament being the most frequently involved structure (21). This corresponds also to our findings. In contrast to our results, Kwok et al. (21) reported more deep endometriotic lesions and a higher incidence of rectal and vaginal endometriosis in patients with unilateral endometriomas. Our analysis indicated that bilateral endometriomas correlated with a higher frequency of coexistent deep endometriosis in the rectovaginal septum.

(#Enzian A), left #Enzian B compartment, and right-sided tubal involvement (#Enzian T). The difference between the results of both studies is hard to explain in light of our current knowledge of endometriosis. One potential reason could be the ethnic differences between the two patient cohorts. The study of Kwok et al. (21) was carried out in China, unlike ours which was carried out in India. It is noteworthy that the study of Kwok et al. (21) did not assess the coexistence of deep and ovarian endometriosis since the inclusion criteria specified patients having both types of the disease.

In general, estimates about the coexistence of deep endometriosis and ovarian endometriomas are lacking in the available literature. Nonetheless, Somigliana et al. (22) reported that deep endometriosis coexists in 92% of cases with at least one different type of endometriosis generally, and it coexists in 53.7% of cases with ovarian endometriomas specifically. In fact, deep endometriosis was found isolated in only 6.5% of cases (22).

The left-sided predominance of endometriosis has been widely documented (13, 23, 24). Additionally, the uterosacral ligament followed by the rectovaginal septum were reported to be the most common location of deep endometriotic lesions (13, 22, 24, 25); which is in line with our results. This asymmetry has been attributed to the clockwise current of the peritoneal fluid induced by the bowel wall’s contraction (26). It is postulated that the regurgitated endometrial cells circulate with the peritoneal fluid and implant in anatomical locations where stasis may be provoked. This principle perfectly explains the left- and right-sided predominance of endometriosis in the pelvis and on the diaphragm, respectively (24, 27). The sigmoid colon is suggested to interrupt the peritoneal current and facilitate the implantation of the endometriotic cells in the left hemipelvis in a similar way to the falciform ligament that facilitates the implantation of endometriosis on the right diaphragmatic dome (26, 28). This notion is supported by the higher incidence of diaphragmatic and thoracic endometriosis on the right than on the left hemidiaphragm (26, 29). Although this postulation provides a plausible explanation of the endometriosis distribution within the body, it remains incapable of determining the origin of endometriosis. A recent study demonstrated that several endometriotic lesions at different body parts share the same clonality and they are more likely to develop from the same origin (30). This may mean that some lesions originate from either the endometrium or other coexisting endometriotic lesions. This postulation is supported by our observation that left-sided endometriomas associate with left-sided deep endometriosis and right-sided endometriomas also correlate to right-sided deep endometriosis.

Finally, it should be noted that the distribution of ovarian endometriomas was found to be influenced by age (28). Bazi et al. (28) reported a significant left-sided predilection of ovarian endometriomas in patients under 35 years old. The authors could not observe the same predilection of ovarian dermoid cysts to be right- or left-sided, indicating that the embryonic origin of endometriosis in younger ages is less probable.

This work has several limitations mostly inherent in the retrospective design of the study and the small sample size. Endometriosis has an important genetic and epigenetic predisposition. Therefore, including patients from the same region and ethnicity limits the generalizability of our results. Furthermore, this study is prone to selection bias due to the single-center design. Considering that the study was carried out at a referral center, the impact of selection bias on the obtained results should not be underestimated. One potential explanation of the high coincidence between endometriomas and deep endometriosis is the referral nature of our center, which may attract more complex cases. Additionally, the lack of a control group without ovarian endometrioma is a weak point because we could not validate the observed association in patients with deep endometriosis but without ovarian endometriomas. Moreover, the hormonal treatment rate is relatively high in our cohort. It is unclear whether hormonal treatments may influence the extension and distribution of endometriosis. Finally, we would like to highlight that despite the relatively large sample size, some subgroup analyses included a small number of patients, which make them underpowered to detect statistically significant differences between the groups, if they actually exist.

## 5 Conclusion

Ovarian endometriomas coexist most frequently with deep endometriosis of the pelvis. Patients with ovarian endometriomas should be preoperatively examined and screened for deep endometriosis in order to guarantee adequate preparation of surgery and complete excision of the disease. The sole surgical treatment of ovarian endometriomas often leads to leaving deep endometriotic lesions behind and consequent incomplete treatment of endometriosis. In patients with either unilateral or bilateral ovarian endometriomas, deep endometriosis is very likely to involve the left parametrium, right parametrium, or rectovaginal septum. Patients with unilateral endometriomas may have deep endometriosis in the ipsilateral hemipelvis. Future research should investigate the distribution of deep endometriosis in different patient populations from various spots of the world and include a control group to validate the observed correlation of ovarian endometriomas on the distribution of deep endometriosis.

## Data Availability

The raw data supporting the conclusions of this article will be made available by the authors, without undue reservation.

## Footnotes

^1^ www.equator-network.org
